# Generation of a Dual-Target, Safe, Inexpensive Microbicide that Protects Against HIV-1 and HSV-2 Disease

**DOI:** 10.1038/s41598-018-21134-1

**Published:** 2018-02-12

**Authors:** Christina Farr Zuend, John F. Nomellini, John Smit, Marc S. Horwitz

**Affiliations:** Department of Microbiology and Immunology, University of British Columbia, Vancouver, British Columbia Canada

## Abstract

HSV-2 infection is a significant health problem and a major co-morbidity factor for HIV-1 acquisition, increasing risk of infection 2–4 fold. Condom based prevention strategies for HSV-2 and HIV-1 have not been effective at stopping the HIV-1 pandemic, indicating that alternative prevention strategies need to be investigated. We have previously developed an inexpensive HIV-1 specific microbicide that utilizes the S-layer mediated display capabilities of *Caulobacter crescentus*, and have shown that recombinant *C*. *crescentus* displaying HIV entry blocking proteins are able to provide significant protection from HIV-1 infection *in vitro*. Here we demonstrate that recombinant *C*. *crescentus* are safe for topical application and describe 5 new recombinant *C*. *crescentus* that provide protection from HIV-1 infection *in vitro*. Further, we demonstrate protection from disease following intravaginal infection with HSV-2 in a murine model using *C*. *crescentus* expressing the anti-viral lectins Cyanovirin-N and Griffithsin, as well as α-1-antitrypsin and indolicidin. Interestingly, *C*. *crescentus* alone significantly reduced HSV-2 replication in vaginal lavage fluid. Protection from HSV-2 disease was strongly associated with early cytokine production in the vaginal tract. Our data support the potential for a dual-target microbicide that can protect against both HIV-1 and HSV-2, which could have an enormous impact on public health.

## Introduction

HIV-1 is a significant global health concern with 2.1 million new infections occurring in 2015^1^. Slightly more than half of the new HIV-1 infections occurred in women, the majority occurring in developing countries where biological factors, sexual violence, a lack of access to prevention services and gender inequalities make young women three times more likely than young men to acquire HIV-1 through sexual transmission^1–4^, suggesting that the development of a female controlled prevention option for HIV-1 is urgently needed.

Herpes Simplex Virus Type 2 (HSV-2), the major cause of genital herpes^5–7^, increases susceptibility to HIV-1 infection. HSV-2 is one of the most common sexually transmitted infections with a prevalence of 10–20% in North America and 30–80% in some developing countries and Sub-Saharan Africa, with higher infection rates in women^8–10^. HSV-2 increases the risk of HIV acquisition by creating breaches in the genital epithelium, and creating a state of chronic inflammation^5–7^. While condoms can provide some protection from HSV-2 infection, there is no highly effective prevention method^7^. Complicating matters, HSV-2 undergoes latency in the nervous system and can periodically reactivate, allowing transmission to occur, even in the absence of clinical symptoms^8,11^. The development of a dual target prevention option for HIV-1 and HSV-2 could be an efficient means to halt infection of both viruses.

Microbicides that are topically applied to the vaginal tract or rectum are an excellent option for female-controlled prevention of HIV-1 and HSV-2 infection^12^. Although over 50 microbicide candidates have been evaluated for HIV-1 prevention, there are currently no commercially available microbicides for HIV-1 or HSV-2^12–14^. A formulation of 1% tenofovir gel applied using a 2 dose before and after sex strategy (BAT24) provided 39% protection from HIV-1 infection and 51% reduction in HSV-2 infections during the CAPRISA 004 clinical trial^2^. However, additional testing of 1% tenofovir gel in the FACTS 001 and VOICE trials resulted in no protective effect^15–17^. Recently, a dapivirine vaginal ring demonstrated 27% efficacy in the ASPIRE trial and 31% efficacy in The Ring Study^18,19^. However, the ring showed low to no efficacy in women under the age of 21^18,19^. The results of these trials indicate there remains an urgent need for microbicide strategies of HIV-1 prevention. We have been developing an HIV-1 microbicide by engineering the freshwater bacteria *Caulobacter crescentus*^20–22^. A *C*. *crescentus* based microbicide could be formulated as a gel or vaginal ring that would be inexpensive to produce and would rely on a per exposure strategy (gel) or provide long-term protection (ring) for prevention of HIV-1 infection. We have created 14 different microbicide candidates that significantly reduce HIV-1 infection *in vitro*^20,22^, with four of these candidates being dual-target against both HIV-1 and HSV-2.

*C*. *crescentus* is a non-pathogenic, Gram-negative freshwater bacterium that is readily isolated from most aquatic and soil sources^23^. The bacterium cannot grow at temperatures above 33 °C or at the salt concentrations found in human blood and tissues^24,25^. *C*. *crescentus* has a surface (S)-layer, a crystalline protein layer composed of a monomer protein that is secreted at very high levels and self-assembles into a lattice on the cell surface^26–28^. We have successfully developed a system to insert foreign protein sequences into the S-layer protein RsaA^28–32^. The foreign proteins often have minimal impact on the secretion and expression of the S-layer, and the proteins retain their function once expressed on the surface of the bacteria^28–32^. As such, S-layer mediated display provides high expression of functional proteins that can be utilized for many biotechnological applications including microbicide development.

We have previously demonstrated that recombinant *C*. *crescentus* displaying MIP1α, CD4, anti-viral lectins and HIV-1 fusion inhibitors are able to provide 22–85% protection from HIV-1 infection *in vitro*, likely through direct interaction with either the viral envelope (CD4, anti-viral lectins, fusion inhibitors) or co-receptor blocking on the target cell (MIP1α)^20,22^. As the anti-viral lectins Cyanovirin-N, microvirin and Griffithsin bind to glycosylation residues on the viral envelope of HIV-1 they are likely able to bind to similar glycosylation patterns on other enveloped viruses, such as HSV-2, and thus may prevent infection. Cyanovirin-N is a virucidal protein isolated from the cyanobacterium *Nostoc ellipsosporum*^33^. Studies have suggested that Cyanovirin-N is able to block HSV-1 cell entry and membrane fusion by interaction with sugar residues on the HSV-1 glycoproteins^34,35^. As HSV-2 has 83% sequence homology with HSV-1, Cyanovirin-N may also be able to prevent HSV-2 infection. Microvirin is a mannose-specific lectin isolated from *Microcystis aeruginosa* that binds to similar carbohydrate structures as Cyanovirin-N with lower toxicity, and may also be able to prevent HSV-2 infection^36,37^. Griffithsin is an anti-viral lectin isolated from the red alga *Griffithsia* sp that has demonstrated anti-viral activity against HSV-2 by blocking cell-to-cell spread of the virus^38^.

Here we describe the generation of 5 new recombinant *C*. *crescentus*: elafin, BmKn2, α-1-antitrypsin, indolicidin and 2X indolicidin multimer. Elafin is a member of the whey acidic protein family that has antiproteolytic, immunomodulatory and antimicrobial properties, and is elevated in cervicovaginal lavage collected from commercial sex workers that are HIV-1 resistant, and affects cell attachment and transcytosis of HIV-1^39,40^. Pretreatment of HIV-1 with elafin has caused a reduction in infection of TZM-bl cells in previous studies^39^. BmKn2 is an antimicrobial peptide that was cloned from scorpion venom and has demonstrated anti-HIV activity *in vitro*, likely by interacting with the viral particle^41^. α-1-antitrypsin is a serpin that plays a role in preventing the fusion of HIV-1 with target cells^42^. Indolicidin is an antimicrobial cationic peptide that has been described to have direct anti-viral activity against HIV-1, thought to be related to its membrane disruptive properties^43,44^. These recombinant *C*. *crescentus* cover a wide spectrum of anti-viral activities, expanding on our previously successful microbicide development. In this study we tested *C*. *crescentus* expressing these recombinant proteins for their ability to provide protection from HIV-1 infection *in vitro* using the TZM-bl cell line and human peripheral mononuclear blood cells (PBMCs) and HSV-2 infection in a mouse model. In addition, Cyanovirin-N, microvirin and Griffithsin^22^, were tested *in vivo* with HSV-2. The five new recombinant *C*. *crescentus* described in this paper provided 21–78% protection from HIV-1 infection *in vitro*. Of the recombinant *C*. *crescentus* tested with HSV-2, four were able to provide protection from HSV-2 disease *in vivo*. While three of the protective recombinant *C*. *crescentus* have previously been suggested to prevent HSV-2 infection by blocking cell-to-cell spread (Griffithsin), preventing entry and membrane fusion (Cyanovirin-N), and disrupting the HSV-2 membrane (Indolicidin), α-1-antitrypsin has not previously been reported to have anti-HSV-2 activity. Protection from HSV-2 disease by Cc-A1AT seems to be mediated by initiation of an early immune response, as this recombinant *C*. *crescentus* led to earlier production of IFNγ, TNF and IL-6 when applied at the time of HSV-2 infection, compared to HSV-2 alone. Finally, we demonstrate that *C*. *crescentus* alone causes little to no inflammatory response in the vagina and so is expected to be safe for repeated topical application. This work describes the continued development of a safe and effective microbicide to prevent infection with both HIV-1 and HSV-2.

## Results

### Expression of Recombinant Proteins on the Surface of C. crescentus

**Figure 1:**
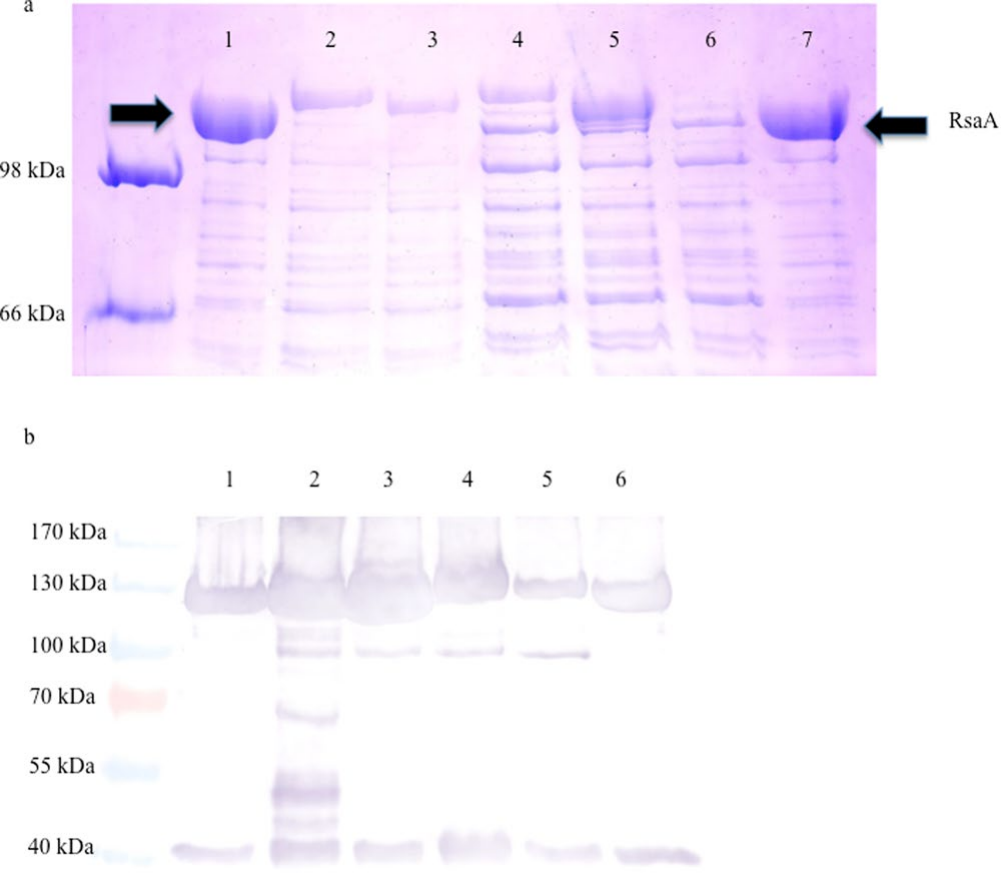
Recombinant S-layer protein gel. Representative Coomassie blue stained 7.5% SDS-PAGE of normalized low pH extracted RsaA protein from *C*. *crescentus* strain JS 4038 containing RsaA plasmids. Arrows indicate the location of the S-layer protein (100 kDa), with size shifts visible to indicate expression of recombinant protein. 1) Cc-Control (no insert); 2) Cc-A1AT; 3) Cc-BmKn2; 4) Cc-Elafin; 5) Cc-Indo; 6) Cc-Indo2; 7) Cc-Control. The image has been brightened and cropped to minimize background. The original image has been provided to *Scientific Reports* and is available from the authors upon request. (**b**) Representative Western blot. RsaA was extracted from *C*. *crescentus* using a low pH method, normalized, run on 7.5% SDS-PAGE using a prestained protein ladder, and RsaA was detected by western blot using an anti-RsaA antibody. 1) Cc-Control (no insert); 2) Cc-A1AT; 3) Cc-BmKn2; 4) Cc-Elafin; 5) Cc-Indo; 6) Cc-Control.

Four proteins, elafin, BmKn2, α-1-antitrypsin and indolicidin, that could prevent HIV-1 attachment or entry were identified from the literature and expressed in the S-layer protein of *C*. *crescentus* as previously described^20–22,28^ (Table 1). The sequence of each expression vector was confirmed before transformation into *C*. *crescentus*. A low pH extraction method, which has been a reliable method to monitor export, assembly and surface attachment of S-layer protein, and SDS-PAGE with Coomassie Brilliant Blue R stain or Western blot was used to assess the expression levels for the chimeric S-layer proteins. All constructs resulted in expression of substantial quantities of protein, and demonstrated small size shifts by protein gel analysis, which, when combined with the sequencing data, provides evidence that the recombinant protein is expressed within the S-layer (Fig. 1a). An indolicidin multimer, containing 2 tandem copies of indolicidin for each one RsaA protein was also constructed. Cc-Indo2 was successfully expressed but produced approximately half the amount of S-layer protein as Cc-Indo. Western blot analysis for *C*. *crescentus* RsaA protein indicated that each recombinant *C*. *crescentus* produced S-layer protein, providing further indication that the bacteria are producing the recombinant proteins (Fig. 1b). While there is variation in the amount of RsaA that is present for each recombinant compared to the Cc-Control, this is unlikely to have a great impact on the ability of each recombinant to prevent HIV-1 infection as a unmodified *C*. *crescentus* expresses 40,000–60,000 copies of the RsaA protein on its’ surface^26,31^, suggesting that even a 50% reduction in RsaA proteins will still lead to substantial expression of the recombinant protein, which is supported by our previous HIV-1 studies^20,22^.

**Table 1:** Overview of recombinant C. crescentus.

Displayed segment	Amino acid sequence of displayed segment	Number of amino acids	Name of Caulobacter display construct	Reference
Alpha-1-antitrypsin	LEAIPCSIPPEFLFGKPFVFLMIEQNTKSPLFMG	34	Cc-A1AT	^42^
Elafin	AQEPVKGPVSTKPGSCPIILIRCAMLNPPNRCLKDTDCPGIKKCCEGSCGMACFVPQ	57	Cc-Elafin	^39,79^
Indolicidin	ILPWKWPWWPWRR	13	Cc-Indo	^43,44^
2xIndolicidin	ILPWKWPWWPWRRASILPWKWPWWPWRR	28	Cc-Indo2	
BmKn2	FIGAIARLLSKIF	13	Cc-BmKn2	^41^

### Protection from *in vitro* HIV-1 infection using S-layer display of Elafin, BmKn2, α-1-Antitrypsin and Indolicidin

**Figure 2:**
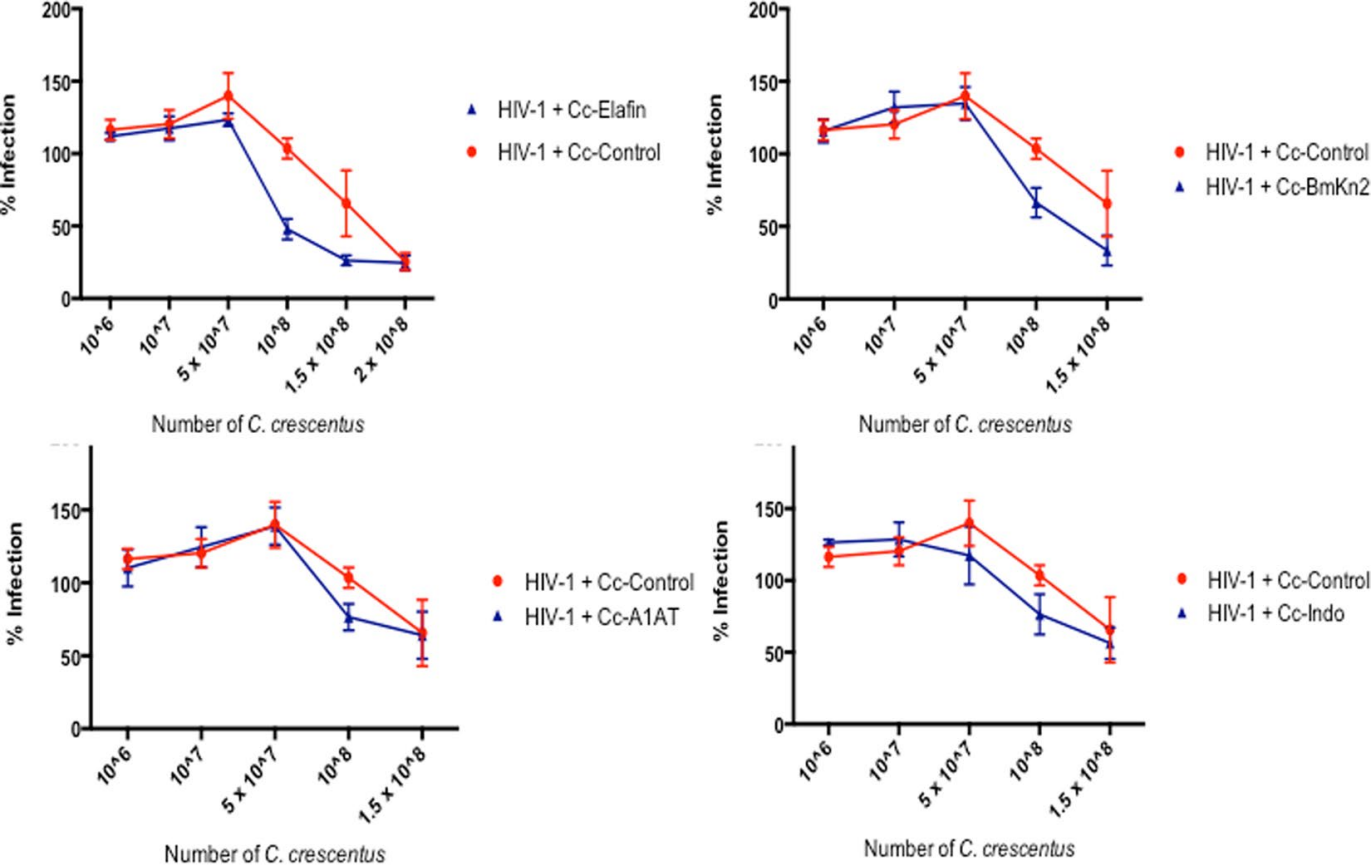
*C*. *crescentus* titration. 10,000 TZM-bl cells were incubated for 48 hours with 200TCID_50_ HIV-1_89.6_ and differing amounts of recombinant *C*. *crescentus* (Cc-Control, Cc-Elafin, Cc-BmKn2, Cc-A1AT or Cc-Indo). HIV-1 infection was measured using a β-galactosidase assay. To minimize assay-to-assay variability in OD_415_ values, the control wells containing HIV-1 + TZM-bl cells with the background of TZM-bl cells subtracted out were set as 100% infection. To obtain the percent infection values for the experimental wells (HIV-1 + TZM-bl cells + Cc-Control or HIV-1 + TZM-bl cells + recombinant *C*. *crescentus*) the OD_415_ values were normalized to the 100% infection (HIV-1 + TZM-bl cells) value. Each experiment was performed in quadruplicate and repeated at least three times.

We have previously demonstrated that 10^8^ recombinant *C*. *crescentus* were sufficient to prevent HIV-1 infection using HIV-1 pseudotyped viruses^20,22^. To confirm that this number of bacteria were sufficient for protection from infection when replication competent HIV-1 was used, we performed a titration of *C*. *crescentus* using 10^6^ – 2 × 10^8^ *C*. *crescentus* (Cc-Control, Cc-Elafin, Cc-BmKn2, Cc-A1AT and Cc-Indo) and HIV-1_89.6_ (Fig. 2). There was no protection from HIV-1 infection with Cc-Control or any of the recombinant *C*. *crescentus* when 10^6^, 10^7^ or 5 × 10^7^ *C*. *crescentus* were added. When 10^8^ *C*. *crescentus* were added the 4 recombinant *C*. *crescentus* provided protection from HIV-1_89.6_ infection whereas the Cc-Control did not. Although protection from HIV-1_89.6_ infection with all 4 recombinants did increase when 1.5 × 10^8^ recombinant *C*. *crescentus* were added the Cc-Control also lowered HIV-1 infection at this dose, which suggests that at high doses *C*. *crescentus* may have some ability to prevent HIV-1 infection, and this was clearly evident when 2 × 10^8^ recombinant *C*. *crescentus* was added, with no difference observed in HIV infection levels between Cc-Control and Cc-Elafin. As the Cc-Control is not predicted to interact with either HIV-1 or the TZM-bl cells, we hypothesize that the large number of *C*. *crescentus* present in such a small cell culture volume (200 μL) is creating a physical barrier, preventing HIV-1 and the TZM-bl cells from interacting. To avoid any impact of the *C*. *crescentus* bacterium itself on HIV-1 prevention 10^8^ recombinant *C*. *crescentus* were used for subsequent experiments.

**Figure 3:**
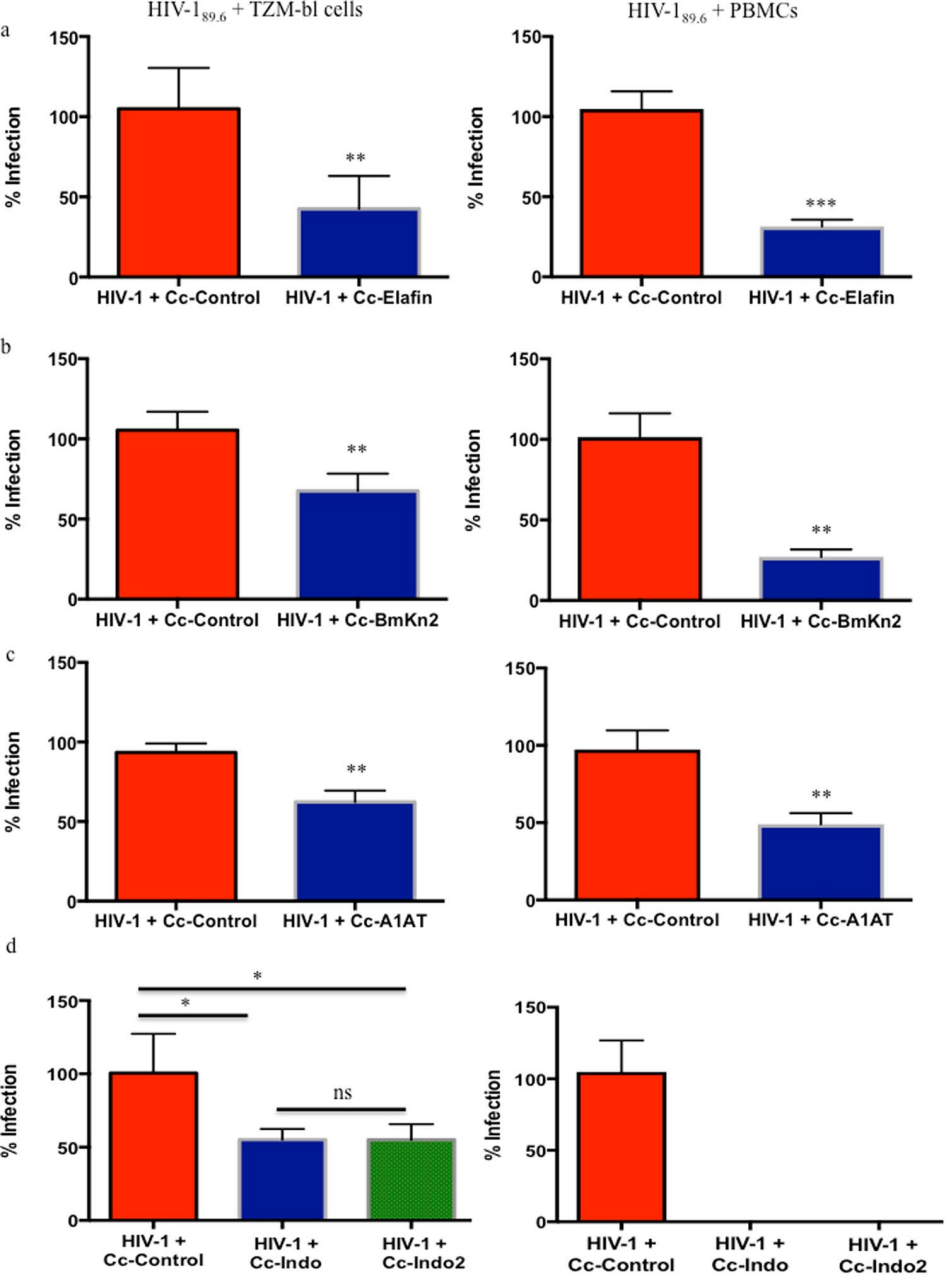
Viral blocking assays. 10,000 TZM-bl cells or PBMCs were incubated for 48 hours with 200TCID_50_ HIV-1_89.6_ and 10^8^ recombinant *C*. *crescentus*. HIV-1 infection was measured using a β-galactosidase assay or p24 ELISA. To minimize assay-to-assay variability in OD_415_ values, the control wells containing HIV-1 + TZM-bl cells with the background of TZM-bl cells subtracted out were set as 100% infection. To obtain the percent infection values for the experimental wells (HIV-1 + TZM-bl cells + Cc-Control or HIV-1 + TZM-bl cells + recombinant *C*. *crescentus*) the OD_415_ values were normalized to the 100% infection (HIV-1 + TZM-bl cells) value. For PBMC experiments data was normalized to wells containing PBMCs + HIV-1_89.6_. Each experiment was performed in quadruplicate and repeated at least three times. Student’s t test or one-way ANOVA with Bonferroni’s correction for multiple comparisons were performed as necessary. Presented statistics represent the comparison between HIV-1 + Cc-Control and HIV-1 + recombinant *C*. *crescentus*. *p < 0.05, **p < 0.01.

Recombinant *C*. *crescentus* displaying elafin, BmKn2, α-1-antitrypsin, indolicidin and an indolicidin multimer individually within the S-layer were incubated with HIV-1_89.6_ for 1 hour before adding TZM-bl cells or PBMCs and HIV-1 infection was measured 48 hours later. Statistically significant protection from HIV-1_89.6_ infection was observed compared to HIV-1 + Cc-Control with Cc-Elafin (57.5% protection TZM-bl cells; 73.4% protection PBMCs), Cc-BmKn2 (36.5% protection TZM-bl cells; 74.3% protection PBMCs), Cc-A1AT (37.7% protection TZM-bl cells; 48.3% protection PBMCs), Cc-Indo (44.8% protection) and Cc-Indo2 (44.9% protection) (Fig. 3a–d). Similar results were obtained with 4 additional HIV-1 strains (Table 2). There was no difference observed between Cc-Indo and Cc-Indo2 in TZM-bl cells. Although Cc-Indo2 contained two tandem copies of indolicidin per each RsaA monomer, the overall expression level of recombinant RsaA on Cc-Indo2 was less than the level of Cc-Indo, which may account for the similar levels of protection. In preliminary experiments, Cc-Indo and Cc-Indo2 provided complete protection of PBMCs from HIV-1_89.6_ infection. Taken together these results suggest that each of these recombinant *C*. *crescentus* are good candidates for further microbicide development.

**Table 2:** Summary of HIV-1 viral blocking data. 10,000 TZM-bl cells or PBMCs were incubated with 200TCID50 HIV-1 and HIV-1 infection was measured 48-72 hours later. Assays were set up in quadruplicate and repeated at least 3 times with the listed viral strains, unless otherwise indicated.

Protein	Name	Live virus TZM-bl cells (mean and range HIV-1 inhibition)^§^	Live virus PBMCs (mean and range HIV-1 inhibition)^#^
Blank Control	Cc-Control	% infection: 110.1% Range 80.8%–137.7%	% infection: 100% Range 99.9%–100%
BmKn2	Cc-BmKn2	38.3% (14.1%–84.3%)	78% (61.2%–83.5%)
α-1-antitrypsin	Cc-A1AT	39.6% (9.9%–87.8%)	56.2% (28.1%–73.6%)
Elafin	Cc-Elafin	44.8% (15.5%–80.4%)	76.5% (57.7%–80.2%)
Indolicidin	Cc-Indolicidin	20.6% (2.2%–52.7%)	100%^*^

^§^Presented mean and range is a summary of data from HIV-1 strains 89.6, BaL, JR-FL, pykJR-CSF.

^#^Presented mean and range is a summary of data from HIV-1 strains 89.6, JR-FL, SF162.

*Performed in quadruplicate with HIV-189.6.

### Immune response following vaginal application of *C*. *crescentus*

**Figure 4:**
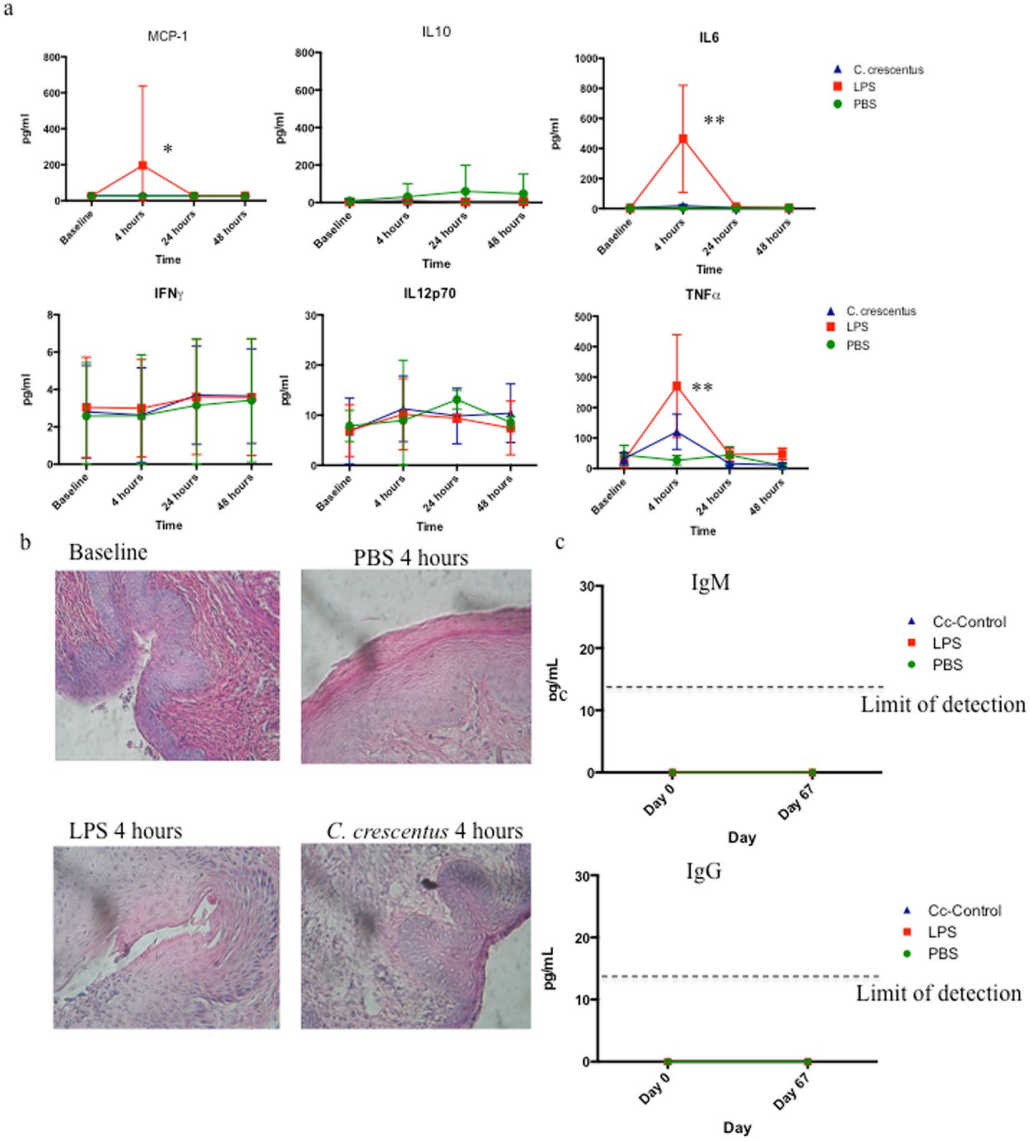
Immune response to intravaginal application of *C*. *crescentus*. (**a**) PBS, 20 μg LPS from *Salmonella enterica serotype Minnesota* or 10^8^ Cc-Control were applied to the vaginal tract of progesterone treated C57Bl/6 mice. Vaginal lavage fluid was collected prior to application then 4, 24 and 48 hours post-application and analyzed by cytometric bead array for mouse inflammatory cytokines. Statistical analysis was performed by two-way ANOVA with Bonferroni’s correction for multiple comparisons. *p ≤ 0.05, **p ≤ 0.01. n = 4–5 (TNF), n = 7–11 (remaining cytokines). (**b**) Hematoxylin and eosin stain vaginal lavage tissue 4 hours after application of PBS, LPS from *Salmonella enterica serotype Minnesota* or *C*. *crescentus*. (**c**) Mice were inoculated intravaginally with 10^8^ *C*. *crescentus* biweekly for 9 weeks before vaginal lavage fluid was collected and analyzed by ELISA for antibodies against *C*. *crescentus*. An anti-RsaA antibody raised in rabbits was used to create a standard curve (limit of detection 14 pg/mL) and values were interpolated from the standard curve.

Previous work by our laboratory^45^ and collaborators^24^ suggested that *C*. *crescentus* is safe for inoculation. However, the mucosal response to *C*. *crescentus* has not been studied. We applied 10^8^ Cc-Control, PBS or LPS isolated from *Salmonella enterica serotype Minnesota* to the vaginal tract of C57Bl/6 mice and collected vaginal lavage fluid from the mice 5 days prior to application, then 4, 24 and 48 hours post-inoculation. Vaginal lavage fluid was analyzed using a cytometric bead array kit for mouse inflammatory cytokines. There was no significant difference between PBS, Salmonella LPS and 10^8^ Cc-Control for IL-10, IFNγ and IL-12p70 (Fig. 4a). Salmonella LPS induced an increase in MCP-1 and IL-6 production at 4 hours post-application compared to both PBS and Cc-Control, but there was no significant difference observed between PBS and Cc-Control (Fig. 4a). Although Cc-Control did cause a small increase in TNF at 4 hours post-application, this difference was not statistically significant compared to PBS and about a third of the amount of TNF that was produced after application of Salmonella LPS, indicating that *C*. *crescentus* does not induce inflammatory cytokines in a manner similar to pathogenic gram negative bacteria (Fig. 4a).

We next investigated whether *C*. *crescentus* application initiated recruitment of immune cells to the vaginal tract. Female C57Bl/6 mice were inoculated intravaginally with PBS, Salmonella LPS or 10^8^ Cc-Control. Four hours later vaginal tissue was excised, fixed and stained with hematoxylin and eosin. There was no evidence of immune cell infiltration to the vaginal tract at 4 hours post-application (Fig. 4b).

Finally, we investigated whether antibodies against *C*. *crescentus* would be produced in the vaginal tract after vaginal application of Cc-Control. As we anticipate that a *C*. *crescentus* microbicide would be used on a semi-regular basis, the mice were inoculated intravaginally bi-weekly for 9 weeks. Vaginal lavage fluid was collected and analyzed for the presence of antibodies against whole *C*. *crescentus* by ELISA, using an anti-RsaA antibody as a positive assay control. No IgG or IgM antibodies against *C*. *crescentus* could be detected in the vaginal fluids (Fig. 4c). Taken together, these results suggest that *C*. *crescentus* may be safe for vaginal application.

### Cyanovirin-N and Griffithsin protect from HSV-2 disease in a murine model

**Figure 5:**
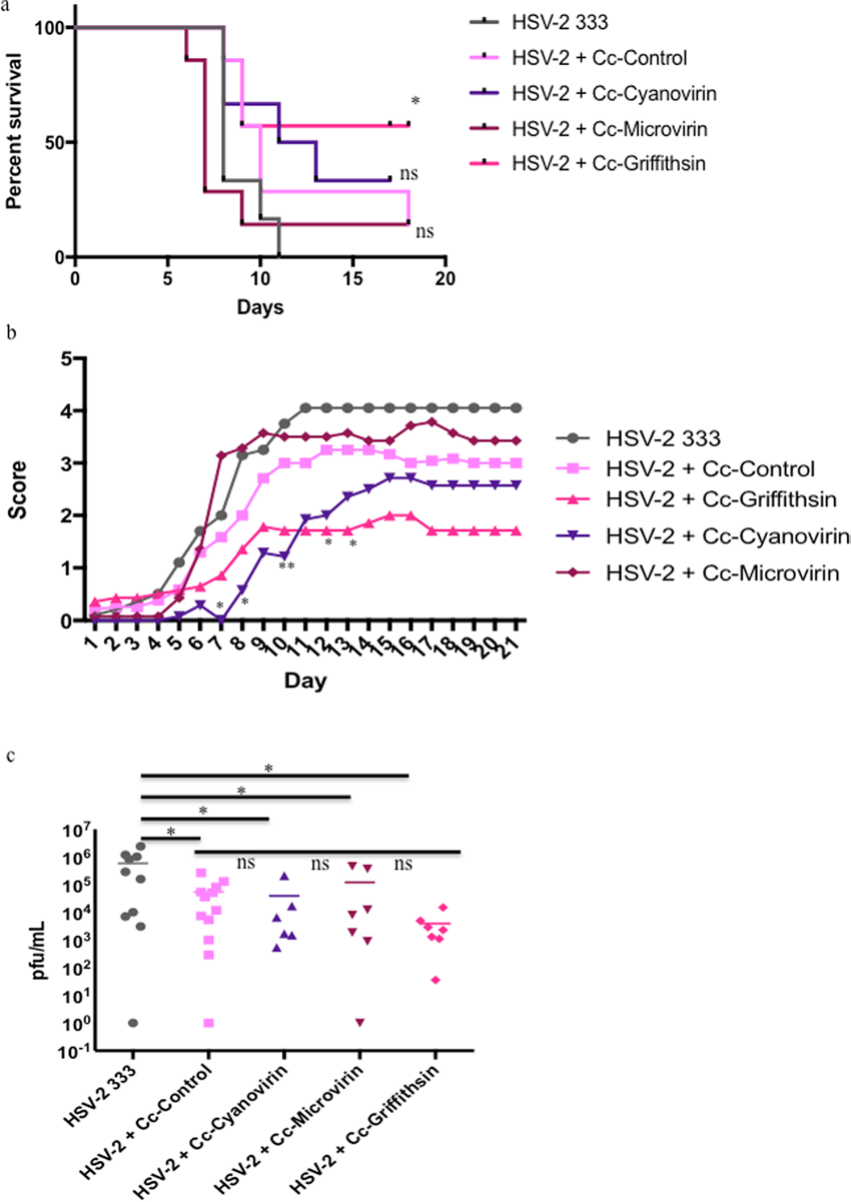
Anti-viral lectins. Progesterone treated C57Bl/6 mice were infected intravaginally with 10^5^ pfu HSV-2 strain 333 in the presence or absence of 10^8^ recombinant *C*. *crescentus*. Mice were scored daily and euthanized if they reached a score ≥4. (**a**) Survival curve. Statistics were performed as a log-rank test. Presented statistics represent the comparison between HSV-2 + Cc-Control and HSV-2 + recombinant *C*. *crescentus*. (**b**) Herpetic infection in the mice was scored daily using a 5-point scale. Data is shown as the average for each treatment group. Two-way ANOVA with Bonferroni’s correction for multiple comparisons were used to calculate statistics. Presented statistics represent the comparison between HSV-2 + Cc-Control and HSV-2 + recombinant *C*. *crescentus*. (**c**) Vaginal lavage fluid was collected on day 2 post-infection. Vaginal lavage fluid was cultured on Vero cells to determine the amount of infectious HSV-2 present. Plaques were counted after 6 days. One-way ANOVA with Bonferroni’s correction for multiple comparisons was used to calculate statistical significance. Presented statistics represent the comparison between HSV-2 and HSV-2 plus each *C*. *crescentus* (p < 0.05) and the comparison between HSV-2 + Cc-Control and HSV-2 plus each recombinant *C*. *crescentus* (p > 0.05). Each data point represents an individual animal. N = 7–12 mice per group. *p < 0.05, **p < 0.01, ns = p > 0.05.

*C*. *crescentus* expressing Cyanovirin-N, microvirin and Griffithsin were previously developed and shown to be able to provide significant protection from HIV-1 infection *in vitro*^22^. Based on published work with HSV-1 and HSV-2 we hypothesized that these recombinant *C*. *crescentus* would also provide protection from HSV-2 infection. To test this we used a murine model of HSV-2 infection. Progesterone treated C57Bl/6 mice were infected intravaginally with HSV-2 in the presence of Cc-Control, Cc-Cyanovirin, Cc-Microvirin or Cc-Griffithsin. All mice that received HSV-2 alone reached the humane endpoint by day 11 post-infection (Fig. 5a). Ten of twelve mice (83%) that were infected with HSV-2 in the presence of Cc-Control reached the humane endpoint by day 9 post-infection (Fig. 5a). Cc-Control application at the time of HSV-2 infection did not significantly enhance survival of the mice compared to HSV-2 alone. The remaining two mice developed mild symptoms of HSV-2 infection between days 6–14 post-infection, indicating that all mice that received Cc-Control were infected, with one of these mice reaching the humane endpoint at day 18. Cc-Griffithsin provided significant protection from HSV-2 mortality compared to both HSV-2 alone and HSV-2 + Cc-Control with 4 of 7 mice (57%) that did not reach the humane endpoint (Fig. 5a). Cc-Cyanovirin protected 3 of 7 mice (43%) from HSV-2 mortality although this was not statistically significant when compared to HSV-2 + Cc-Control (Fig. 5a). Cc-Microvirin provided no protection from HSV-2 infection, with 6 of 7 mice reaching the humane endpoint at the same time as mice that received HSV-2 alone (Fig. 5a). Cc-Griffithsin application at the time of HSV-2 infection led to a significant decrease in severity of HSV-2 symptoms when compared to both HSV-2 + Cc-Control and HSV-2 alone (Fig. 5b). Mice that received Cc-Cyanovirin had a statistically significant delay in developing symptoms of HSV-2 infection up to day 12 post-infection when compared to both HSV-2 alone and HSV-2 + Cc-Control (Fig. 5b). Cc-Microvirin did not have an impact on severity of HSV-2 symptoms (Fig. 5b). There was no statistically significant difference in the time to development or severity of HSV-2 symptoms between mice that received HSV-2 alone and Cc-Control (Fig. 5b).

Interestingly, when compared to HSV-2 alone, all recombinant *C*. *crescentus* tested, including Cc-Control and Cc-Microvirin, which did not prevent development of HSV-2 symptoms, provided a significant reduction of approximately 1 log in viral load in the vaginal lavage fluid at day 2 post-infection (Fig. 5c). Despite this significant decrease in HSV-2 replication, Cc-Control and Cc-Microvirin did not significantly increase survival or decrease herpetic scores in the mice when compared to those that received HSV-2 alone, suggesting that there may be some non-specific anti-viral effects of recombinant *C*. *crescentus*. This could potentially be due to the bacteria providing a physical barrier that makes it more difficult for HSV-2 to interact with epithelial cells to cause infection. While the same bacterial barrier would be present in mice that received Cc-Griffithsin or Cc-Cyanovirin, the additional ability of these recombinant proteins to prevent cell-to-cell spread of HSV-2 (Cc-Griffithsin) or HSV-1 entry and membrane fusion (Cc-Cyanovirin)^34,38^ could account for the improved survival and lower herpetic scores observed in the mice that received these recombinant *C*. *crescentus*.

### Prevention of in vivo HSV-2 disease by Cc-A1AT and Cc-Indo

All of the new inhibitors developed for this paper were tested *in vivo* for protection against HSV-2 infection. Indolicidin has been demonstrated to reduce HSV-2 infectivity *in vitro*^43^ and elafin has been demonstrated to have *in vitro* and *in vivo* anti-viral activity against HSV-2^45^. α-1-antitrypsin is a serpin antiprotease which are key players in the regulation of inflammatory responses^46^, so α-1-antitrypsin may be able to protect from HSV-2 infection by regulating inflammation in the vaginal tract. The exact anti-viral mechanism of BmKn2 has yet to be elucidated, and it was possible it may have broad anti-viral activity.

**Figure 6:**
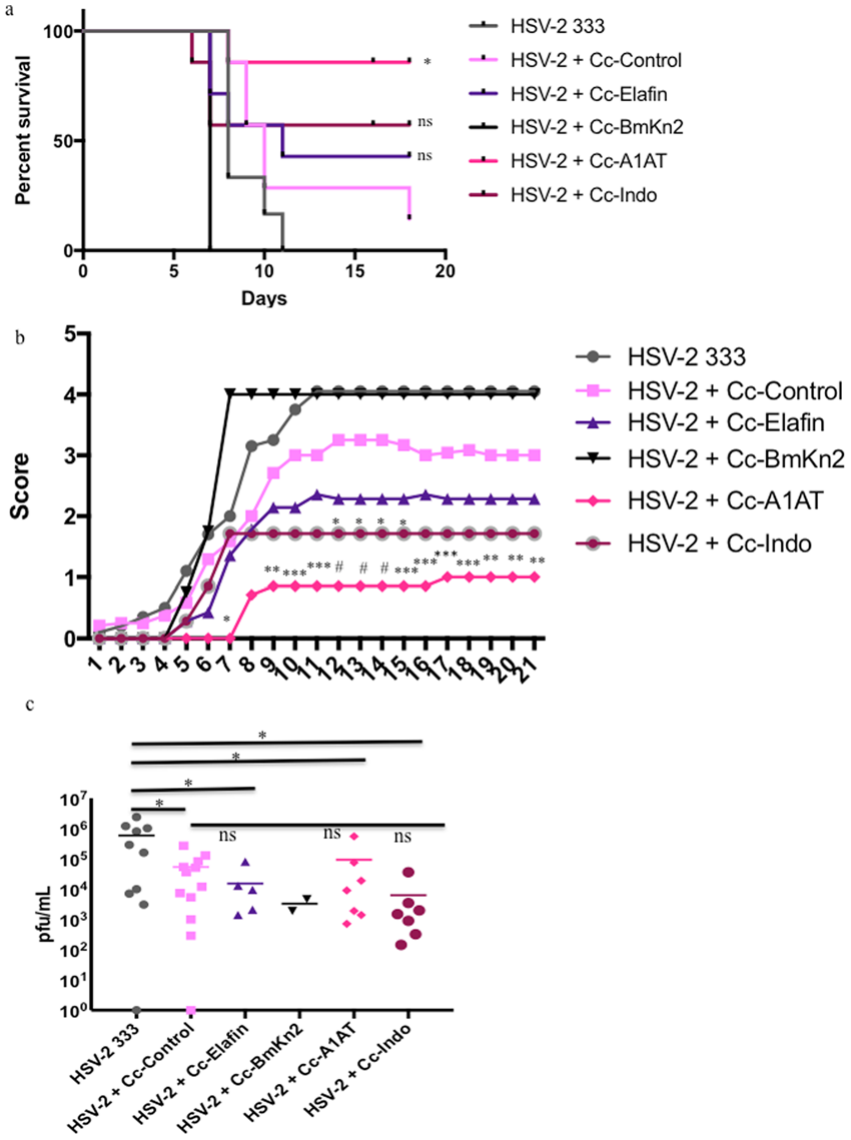
HSV-2 murine model with new inhibitors. Progesterone treated C57Bl/6 mice were infected intravaginally with 10^5^ pfu HSV-2 strain 333 in the presence or absence of 10^8^ recombinant *C*. *crescentus*. Mice were scored daily and euthanized if they reached a score ≥4. (**a**) Survival curve. Statistics were performed as a log-rank test. Presented statistics represent the comparison between HSV-2 + Cc-Control and HSV-2 + recombinant *C*. *crescentus*. (**b**) Herpetic infection in the mice was scored daily using a 5-point scale. Data is shown as the average for each treatment group. Two-way ANOVA with Bonferroni’s correction for multiple comparisons were used to calculate statistics. Presented statistics represent the comparison between HSV-2 + Cc-Control and HSV-2 + recombinant *C*. *crescentus*. (**c**) Vaginal lavage fluid was collected on day 2 post-infection. Vaginal lavage fluid was cultured on Vero cells to determine the amount of infectious HSV-2 present. Plaques were counted after 6 days. One-way ANOVA with Bonferroni’s correction for multiple comparisons was used to calculate statistical significance. Presented statistics represent the comparison between HSV-2 and HSV-2 plus each *C*. *crescentus* (p < 0.05) and the comparison between HSV-2 + Cc-Control and HSV-2 plus each recombinant *C*. *crescentus* (p > 0.05). Each data point represents an individual animal. N = 7–12 mice per group. *p < 0.05, **p < 0.01, ***p < 0.001, #p < 0.0001, ns = p > 0.05.

Cc-A1AT provided significantly improved survival following HSV-2 infection with 6 of 7 mice (86%) surviving challenge compared to both HSV-2 alone and to HSV-2 + Cc-Control (Fig. 6a). Cc-Indo protected 4 of 7 mice (57%) from HSV-2 mortality but this was not statistically significant (Fig. 6a). Cc-Elafin protected 3 of 7 mice (43%) from HSV-2, however this was not statistically significant (Fig. 6a). In preliminary experiments mice that received HSV-2 + Cc-BmKn2 rapidly reached the humane endpoint, at an earlier day post-infection than HSV-2 alone, indicating that Cc-BmKn2 was not a good candidate for HSV-2 prevention and additional experiments were not undertaken (Fig. 6a). There was a slight delay in the development of symptoms and a significant reduction in symptom severity with the mice that received HSV-2 + Cc-A1AT when compared to both HSV-2 alone and HSV-2 + Cc-Control (Fig. 6b). There was a significant decrease in severity of HSV-2 scores for mice that received Cc-Indo compared to HSV-2 alone, and on days 12–15 post-infection when compared to HSV-2 + Cc-Control (Fig. 6b). Although the disease score for mice receiving Cc-Elafin was lower, this was not statistically significant. When vaginal lavage fluid was analyzed for viral load, there was a significant reduction of approximately 1 log in viral load between HSV-2 alone and HSV-2 plus Cc-Control; Cc-Elafin; Cc-A1AT; and Cc-Indo (Fig. 6c). As all recombinant *C*. *crescentus* lowered viral load but not all prevented HSV-2 infection this suggests that there are non-specific anti-viral effects, and that those recombinants that protect from infection use an additional mechanism to provide protection. Based on previous studies, Cc-Indo is likely disrupting the viral membrane of HSV-2 to prevent infection^43,44^. However, Cc-A1AT was not anticipated to have direct anti-HSV-2 activity so further studies were undertaken to for further investigation.

### Cc-A1AT produces early cytokine responses in the vaginal tract following HSV-2 infection

Given that HSV-2 viral loads in the vaginal lavages were similar between the Cc-Control and each recombinant *C*. *crescentus*, but that not all *C*. *crescentus* improved survival and lowered HSV-2 symptom severity, we hypothesized that the *C*. *crescentus* themselves were providing non-specific protection by interfering with the ability of HSV-2 to interact with epithelial cells, similar to the effects observed with high amounts of bacteria added to *in vitro* HIV-1 assays (Fig. 2a). While proposed mechanisms of protection have been elucidated for Cc-Cyanovirin, Cc-Griffithsin and Cc-Indo, Cc-A1AT was not anticipated to have anti-HSV-2 ability as it interacts specifically with gp41 present on HIV-1. However, α-1-antitrypsin is a serpin antiprotease that can mediate inflammatory responses, so it is possible that the presence of α-1-antitrypsin at the time of HSV-2 infection could mediate a protective immune response. Preliminary *in vitro* viral blocking assays with Vero cells suggested that direct interaction with HSV-2 or target cells was an unlikely mechanism of action for Cc-A1AT as there was no impact on development of HSV-2 plaques with Cc-A1AT. We investigated the production of inflammatory cytokines following Cc-A1AT application in the presence or absence of HSV-2 to determine if the protection from HSV-2 infection was potentially immume mediated. Mice were inoculated intravaginally with HSV-2, Cc-Control, recombinant *C*. *crescentus*, HSV-2 + Cc-Control, or HSV-2 plus recombinant *C*. *crescentus*. Vaginal lavage fluid was collected 4 days prior to inoculation then at 4, 24 and 48 hours post-inoculation and analyzed by cytometric bead array for mouse inflammatory cytokines. Cc-A1AT was analyzed because it was the most successful at preventing HSV-2 disease, Cc-Griffithsin because it protected approximately half of the mice from HSV-2 disease, Cc-Control because it did not protect from HSV-2 infection and Cc-BmKn2 because it may enhance HSV-2 infection.

**Figure 7:**
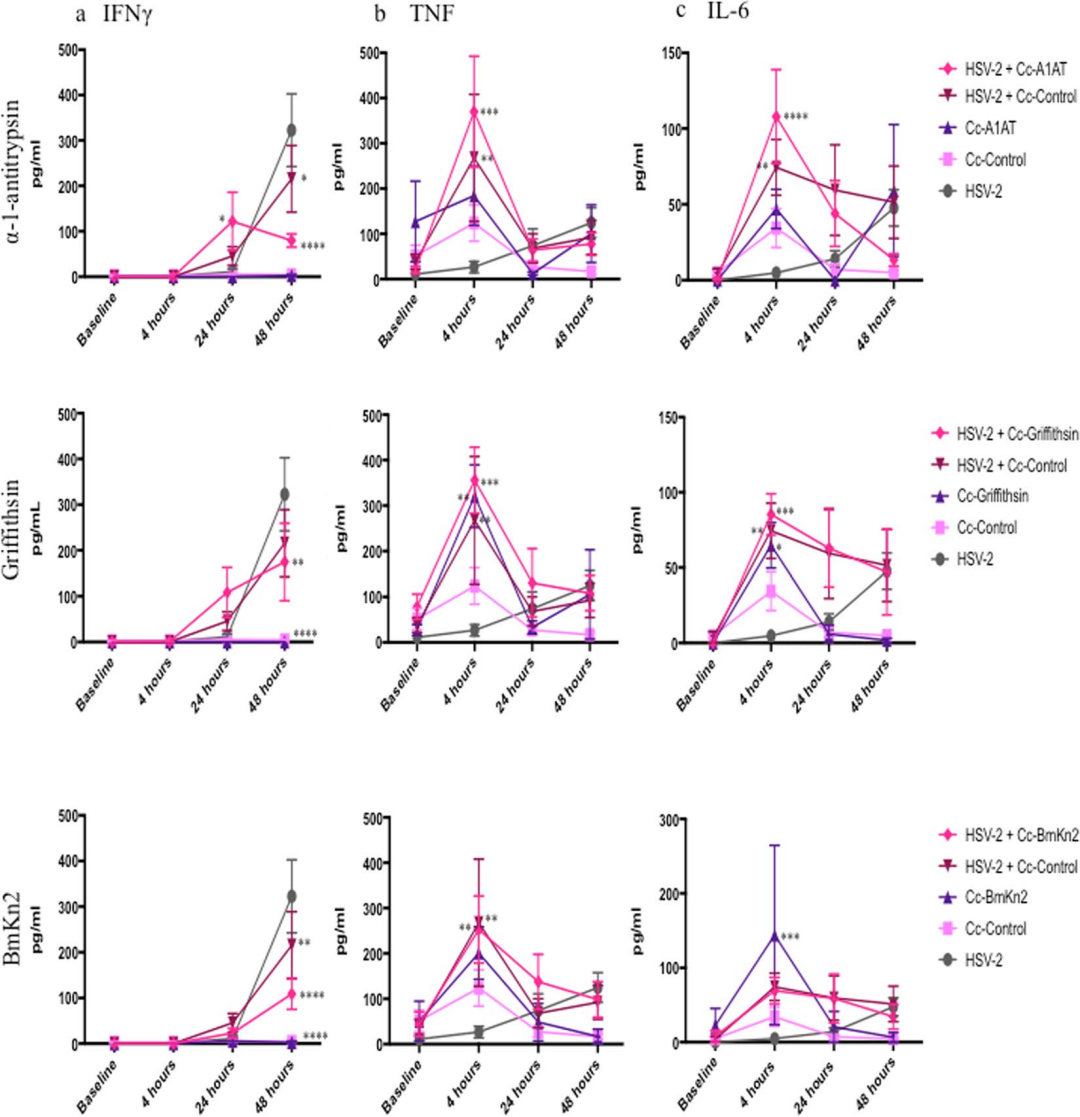
Cytokine analysis after HSV-2 +/− *C*. *crescentus*. Progesterone treated C57Bl/6 mice were infected intravaginally with 10^5^ pfu HSV-2 strain 333 in the presence or absence of individual recombinant *C*. *crescentus* Cc-Control, Cc-BmKn2, Cc-Griffithsin or Cc-A1AT. Vaginal lavage fluid was collected prior to infection then 4, 24 and 48 hours post-infection and analyzed in a cytometric bead array for mouse inflammatory cytokines. (**a**) IFNγ (**b**) TNF (**c)** IL-6. N = 4–11 mice per group. For each cytokine statistics were performed using two-way ANOVA with Bonferroni’s correction for multiple comparisons. Presented statistics represent the comparison between HSV-2 and each experimental group. All other statistical comparisons were not significant. *p < 0.05, **p < 0.01, ***p < 0.001, ****p < 0.0001.

At 24 hours post-infection, mice that received HSV-2 + Cc-A1AT had significantly increased (11 fold more) IFNγ in the vaginal lavage compared to mice that were infected with HSV-2 alone (Fig. 7a). There was 2.7 fold more IFNγ when mice received HSV-2 + Cc-A1AT compared to HSV-2 + Cc-Control, although this was not statistically significant. In comparison, although mice that received HSV-2 + Cc-Griffithsin had elevated IFNγ in the vaginal tract at 24 hours post-infection, this was not significantly greater than HSV-2 alone or HSV-2 + Cc-Control, and about a third the amount of IFNγ that the HSV-2 + Cc-A1AT mice produced (Fig. 7a). Furthermore, mice that received HSV-2 + Cc-BmKn2 or HSV-2 + Cc-Control had no increase in IFNγ in the vaginal tract at 24 hours post-infection (Fig. 7a). At 4 hours post-infection, all recombinant *C*. *crescentus* tested, including Cc-Control, caused a significant increase in the amount of TNF present in the vaginal lavage when given at the same time as HSV-2 (Fig. 7b). Cc-A1AT and Cc-Griffithsin produced similar levels of TNF. Although the levels of TNF produced by either HSV-2 + Cc-A1AT or HSV-2 + Cc-Griffithsin were not statistically significant compared to HSV-2 + Cc-Control, both of these recombinant *C*. *crescentus* led to production of a third more TNF than either HSV-2 + Cc-Control and HSV-2 + Cc-BmKn2, and ten times more TNF than HSV-2 alone. Furthermore, HSV-2 + Cc-A1AT produced significantly more IL-6 at 4 hours post-infection than HSV-2 alone (Fig. 7c). Although the comparison between HSV-2 + Cc-C-A1AT and HSV-2 + Cc-Control was not statistically significant, HSV-2 + Cc-A1AT produced 32% more IL-6 than HSV-2 + Cc-Control at this time point. HSV-2 + Cc-Griffithsin also produced significantly more IL-6 at 4 hours post-infection compared to virus alone, although this was about 20% less IL-6 than what was produced by HSV-2 + Cc-A1AT, and not statistically significant when compared to HSV-2 + Cc-Control (Fig. 7c). Although HSV-2 + Cc-BmKn2 did cause a slight elevation in IL-6 at 4 hours post-infection, this was not statistically significant (Fig. 7c). Cc-A1AT alone did not produce significantly more IFNγ, TNF or IL-6 in the vaginal tract compared to Cc-Control alone (Fig. 7). There was no significant difference between levels of IL-12p70, IL-10 and MCP-1 for HSV-2 compared to HSV-2 + Cc-A1AT, or HSV-2 + Cc-Griffithsin at any of the time points (Supplementary Figure 1). Taken together, these results suggested that Cc-A1AT could protect mice from HSV-2 infection by inducing an earlier cytokine response in the vaginal tract during HSV-2 infection, with IFNγ production 24 hours earlier, TNF about 20 hours earlier, and IL-6 produced 2 days earlier than HSV-2 alone. Although the comparisons between HSV-2 + Cc-A1AT and HSV-2 + Cc-Control for each of these time points were not statistically significant, we hypothesize that the increases in IFNγ (2.7 fold), TNF (1.4 fold) and IL-6 (1.4 fold) compared to HSV-2 + Cc-Control are biologically significant and contribute to the significant reduction in HSV-2 symptom severity and significant increase in survival observed in mice that received HSV-2 + Cc-A1AT compared to HSV-2 alone and HSV-2 + Cc-Control.

## Discussion

In this paper we have continued our development of a *C*. *crescentus* based microbicide. We describe the creation of five new recombinant *C*. *crescentus* and demonstrate that these recombinant bacteria are able to provide 36–58% protection from HIV-1 infection *in vitro*. Furthermore, three of the anti-HIV *C*. *crescentus* that we previously published and the five new candidates presented here were tested for their ability to provide protection from HSV-2 disease. We found that *C*. *crescentus* expressing α-1-antitrypsin, Griffithsin, Cyanovirin-N and indolicidin are able to provide some protection of mice from vaginal HSV-2 disease. This seemed to be independent of viral load as *C*. *crescentus* alone was able to cause a significant reduction in HSV-2 in vaginal fluid. When we measured cytokine production in the vaginal fluid after inoculation with HSV-2 in the presence or absence of recombinant *C*. *crescentus*, we found that Cc-A1AT induced an earlier production of IFNγ, TNF and IL-6 compared to virus alone. In particular, the amount of IFNγ produced at 24 hours post-infection seemed to correlate with protection, with Cc-A1AT producing 15 times more IFNγ than virus alone and protecting 86% of mice, Cc-Griffithsin producing 10 times more IFNγ and protecting 57% of mice, Cc-Control providing no protection and producing 4 times more IFNγ and Cc-BmKn2 providing no protection and producing double the amount of IFNγ. Although the comparison between HSV-2 + Cc-Control and HSV-2 + Cc-A1AT was not statistically significant, there was 2.7 times more IFNγ, 1.4 times more TNF and 1.4 times more IL-6 produced with HSV-2 + Cc-A1AT. We propose that this increase in cytokine production is biologically significant and that this earlier and greater cytokine response could initiate an earlier recruitment of immune cells to the vaginal tract, which may be able to contain the viral infection before the virus has the chance to cause pathology, although further studies are warranted to investigate this. Although both HSV-2 + Cc-Control and HSV-2 + Cc-BmKn2 do lead to an increase in IFNγ, perhaps it is not enough to reach the threshold necessary to protect the mice from HSV-2 infection.

Our data has suggested that Cc-A1AT provides protection from HSV-2 disease by inducing an earlier immune response in the vaginal tract, possibly resulting in earlier immune cell recruitment to the vaginal tissue to control HSV-2 infection. This cytokine production could be problematic for HIV-1 prevention, as genital inflammation has been shown to increase HIV-1 acquisition^47–53^. In particular, in CAPRISA 002 increased levels of proinflammatory cytokines were associated with increased HIV-1 infection, and this finding was validated in the CAPRISA 004 clinical trial, with women having an elevation of three or more proinflammatory cytokines in the cervicovaginal lavage at a 3-fold increased risk of HIV-1 acquistion^54,55^. However, we do not anticipate this to be problematic with a *C*. *crescentus* based microbicide based on our data. First, when Cc-A1AT alone was applied to the vaginal tract of mice there was no production of inflammatory cytokines, indicating the recombinant bacterium alone does not induce inflammation. In addition, preliminary data investigating *in vivo* protection from vaginal HIV-1 infection in humanized bone marrow-liver-thymus (BLT) mice with Cc-A1AT indicated that protection from HIV-1 infection occurred (*Farr Zuend et al*., *submitted)*. This suggests that it is the combination of Cc-A1AT plus HSV-2 that is responsible for the earlier cytokine response, and that in the absence of HSV-2 there is no concern for an increased immune response in the vaginal tract.

In these studies the recombinant *C*. *crescentus* was applied at the time of HSV-2 infection, which may not be feasible in a “real world” situation. We have undertaken preliminary studies in which Cc-A1AT or Cc-Control were applied to the vaginal tract 2 hours prior to HSV-2 infection and found that similar levels of protection from HSV-2 disease were maintained, with 100% of mice that received HSV-2 + Cc-Control reaching the humane endpoint and only 33% of mice that received HSV-2 + Cc-A1AT reaching the humane endpoint. Similarly, scores of HSV-2 infection were reduced by more than two-fold in mice that received HSV-2 + Cc-A1AT, indicating that recombinant *C*. *crescentus* do not need to be applied at the time of exposure for protection from HSV-2 disease.

Interestingly, *C*. *crescentus* alone had some ability to lower HIV-1 infection when used at high doses, and was able to provide a 1 log reduction in HSV-2 viral load in vaginal lavage. While we have hypothesized that this may be an indirect method of prevention, with the presence of large amounts of *C*. *crescentus* providing a non-specific barrier that can lower the ability of HIV-1 or HSV-2 to infect cells further studies are necessary to determine if *C*. *crescentus* itself exerts some sort of non-specific anti-viral effect. If *C*. *crescentus* has some inherent anti-viral activity this would further its suitability and desirability as a microbicide candidate as both the recombinant protein, which binds directly to the virus or target cells, as well as the bacteria itself could work together to enhance protection from infection.

HSV-2 infection is a major risk factor for HIV-1 acquisition, leading to a 2–4 fold increase in HIV-1 infection^56^. The use of condoms, disclosure of serostatus and anti-viral therapy are only 50% effective at preventing HSV-2 infection^7^. Several attempts have been made to develop a vaccine against HSV-2, with no reports of success in people^7,56–60^. Furthermore, HIV-1 positive women have impaired mucosal immunity to HSV-2 infection and diminished anti-HSV activity of cervico-vaginal secretions^6^. There is currently no vaccine for HIV-1, and prevention efforts focused on condom use have not been successful in protecting women from HIV-1 infection. A dual-target microbicide could have a major impact on women’s sexual health.

Both HIV-1 and HSV-2 cause significant morbidity each year. By preventing HSV-2 infection we may be able to prevent numerous HIV-1 infections. A microbicide that is targeted to two sexually transmitted viruses could have a huge impact on the HIV-1 epidemic, and could prevent HSV-2 infections, having an enormous impact on public health. We have previously demonstrated that *C*. *crescentus* is easily modifiable to express a wide variety of anti-HIV-1 proteins, and that these recombinant *C*. *crescentus* can provide significant protection from HIV-1 infection *in vitro*. We have built on our previous findings to show preliminary safety data for a *C*. *crescentus* based microbicide, creation of new recombinant *C*. *crescentus*, protection from HIV-1 infection, and that we have created a dual-target microbicide with several candidates protecting against both HIV-1 and HSV-2 infection.

## Materials and Methods

### Cell Lines and Viruses

Vero cells were obtained from the American Tissue Culture Collection and maintained in MEM supplemented with 7.5% fetal bovine serum (Gibco), 5 mM sodium pyruvate, 1X MEM non-essential amino acids, 1 U/mL penicillin and 1 μg/mL streptomycin (Gibco). The following reagents were obtained through the NIH AIDS Reagent Program, Division of AIDS, NIAID, NIH: TZM-bl cells from Dr. John C. Kappes, Dr. Xiaoyun Wu and Tranzyme Inc^61–65^; 174xCEM cells from Dr. Peter Cresswell^66^; HIV-1_89.6_ from Dr. Ronald Collman^67^; pYK-JRCSF (Cat# 2708) from Dr. Irvin SY Chen and Dr. Yoshio Koyanagi^68–70^ and was a gift from Dr. Zabrina Brumme (Simon Fraser University); SF162 from Dr. Jay Levy^71^; BaL from Dr. Suzanne Gartner, Dr. Mikulas Popovic and Dr. Robert Gallo^72,73^; JR-FL from Dr. Irvin Chen^69,74,75^. HIV-1_89.6_ was propagated in 174xCEM cells with 7.5 μg/mL DEAE-dextran (Sigma) and was harvested at peak CPE between 7–10 days. HIV-1_pykJR-CSF_ was propagated in 293 T cells using Lipofectamine 2000 (Invitrogen) according to the manufacturer’s instructions. HIV-1_JR-FL_ was propagated in 5 × 10^6^ PHA-stimulated PBMCs using 10 μg/mL polybrene (Hexadimethrine bromide, Sigma). Virus was harvested on days 5, 6 and 7 post-infection. HIV-1_BaL_ was propagated in PHA-stimulated PBMCs with 7.5 μg/mL DEAE-dextran for 10 days. HIV-1_SF162_ was propagated in PHA-stimulated PBMCs with 10 μg/mL polybrene for 10 days. HSV-2 (strain 333) was a gift from Dr. Charu Kaushic, McMaster University. HSV-2 was propagated and titred on Vero cells. Briefly, Vero cells were grown to 70% confluence then infected with MOI 0.05 HSV-2. Viral supernatant was harvested when CPE reached 60%. Viral stocks were determined by plaque assay. Virus was serially diluted in serum-free media and incubated with Vero cells for 1 hour at 37 °C with shaking, then 2% agar (Sigma) and 2X MEM (Gibco) supplemented with 10% FBS was added at a 1:1 ratio. Plates were incubated at 37 °C for 6 days before fixing with 25% paraformaldehyde and staining with 1% Crystal violet.

#### Primary cells

Whole blood was collected and processed using Lymphoprep (Stemcell Technologies Inc). PBMCs were grown in RPMI 1640 supplemented with 20% FBS. All procedures were approved by the University of British Columbia Clinical Research Ethics Board certificate H12–02480.

### Preparation of *C*. *crescentus* Displaying Chimeric S-layer Proteins

*C*. *crescentus* strain JS4038 was used for all experiments. Gene segments were synthesized by Integrated DNA Technologies, Inc. (Coralville, Iowa) with codon usage adapted for *C*. *crescentus*. See Table 1 for the amino acid sequences. The synthesized DNA segments specified *Bgl*II and *Spe*I restriction sites on the 5′ side and an *Nhe*I site on the 3′ end to facilitate directional cloning into p4BRsaA(723)/GSCC digested with *Bgl*II and *Nhe*I^29^. p4BRsaA(723)/GSCC is identical to p4ARsaA(723)/GSCC except an extra, irrelevant *Bam*H1 site has been removed by digesting with the enzyme, filling in ends with Klenow DNA polymerase and blunt self ligation^26^. The 2X indolicidin was made in a fashion similar to other tandem repeat multimer clones^29^. Inserted sequences were confirmed by DNA sequencing before transfer to *C*. *crescentus* by electroporation.

### Caulobacter crescentus

*C*. *crescentus* were grown in PYE medium (0.2% peptone, 0.1% yeast extract, 0.01% CaCl_2_, 0.02% MgSO_4_) with 2 μg/mL chloramphenicol to an optical density at 600 nm of approximately 1 (3 × 10^9^ cells/ml). Cells were centrifuged and suspended in sterile water three times and cell density was adjusted to 5 × 10^8^ or 5 × 10^9^ cells/mL for experiments.

### Protein Analysis

*C*. *crescentus* were grown to an optical density at 600 nm of approximately 1 (3 × 10^9^ cells/mL) and protein was prepared from equal numbers of bacterium. S-layer proteins were prepared by a low-pH extraction method^76^. S-layer proteins were visualized using sodium dodecyl sulfate-polyacrylamide gel electrophoresis (SDS-PAGE) using 7.5% separating gels. For Coomassie Brilliant Blue R stained gels the Coomassie Bio-Rad SDS-PAGE standards, low range (Bio-Rad) was used. The image has been brightened and cropped to minimize background. The original image has been provided to *Scientific Reports* and is available from the authors upon request. For immuno-blotting, proteins were transferred to BioTrace NT nitrocellulose membranes (Pall Life Sciences). Membranes were blocked with Tris-buffered saline (TBS)–milk, (20 mM Tris base, 150 mM NaCl, pH 7.4, containing 3% [wt/vol] nonfat milk) and incubated with appropriate primary antibodies in TBS-milk. Membranes were then washed (3X) in TBS-milk, then incubated with appropriate Horse Radish peroxidase (HRP) conjugated secondary antibodies in TBS-milk. Blots were washed with TBS and visualized with 4-chloro-1-naphthol as previously described^27,77^. Immunoblots to identify chimeric RsaA proteins were done using a rabbit anti-RsaA antibody (1:10,000 dilution) and goat anti-rabbit HRP secondary antibody (1:10,000 dilution). A lane containing Fermentas Page ruler prestained protein ladder (Thermo-Fisher)^26,78^ was used as a size standard.

### HIV-1_89.6_ Viral Blocking Assays

*C*. *crescentus* were grown and prepared as described above immediately prior to use. All experiments were performed in quadruplicate wells and repeated at least three times. 200TCID_50_ was determined as previously described^22^ and used for all virus blocking assays. For titrations 10^6^, 10^7^, 5 × 10^7^, 10^8^, 1.5 × 10^8^ or 2 × 10^8^ *C*. *crescentus* were added to each well and 1 × 10^8^ *C*. *crescentus* were added for viral blocking experiments. The virus and *C*. *crescentus* constructs were incubated for 1 hour at 37 °C before adding 10,000 TZM-bl cells or PBMCs and 75 μg/mL DEAE-dextran. After an overnight incubation at 37 °C and 5% CO_2_ the plates were centrifuged at 800 rpm for 5 minutes and medium was changed in all wells (PBMCs only). After an additional 24–48 hours the level of infection was determined by β-galactosidase assay kit (TZM-bl cells) or p24 ELISA (ZeptoMetrix Corporation and ProSci Incorporated) according to the manufacturer’s instructions (PMBCs). For β-galactosidase assays data are presented and determined as a percentage of infection of the TZM-bl + HIV-1 wells with the background from uninfected TZM-bl cells subtracted. p24 ELISA data is normalized to infection of the untreated control wells with PBMCs + HIV-1 set as 100%.

### Ethics Statement

All animal work was performed under strict accordance with the regulations of the Canadian Council for Animal Care. The protocol was approved by the Animal Care Committee of the University of British Columbia (certificate number A12–0245).

### Mice

Female, 6 week old C57Bl/6 mice were purchased from The Jackson Laboratory and bred and maintained in the rodent facility at the University of British Columbia for use in all experiments.

### Cytokine Analysis

Mice were injected subcutaneously with 2 mg Medroxyprogesterone 17-acetate (Sigma) 4 days prior to intravaginal inoculation with 20 μg LPS from *Salmonella enterica serotype Minnesota* (Sigma), HSV-2 alone, 10^8^ recombinant *C*. *crescentus* alone, or HSV-2 plus *C*. *crescentus*. Vaginal inoculation was performed in a volume of 20 μL using a sterile p200 pipette. Vaginal lavage was performed 4 days prior to and 4, 24 and 48 hours post-inoculation by pipetting twice consecutively with 30 μL PBS. Vaginal lavage fluid was centrifuged at 400 × g and the supernatant was stored at −80 °C until analysis. 25 μL vaginal lavage fluid was analyzed using a Cytometric Bead Array kit for Mouse Inflammation (BD Biosciences, Catalog No. 552364) that detects mouse IFNγ, TNF, IL-6, IL-10, MCP-1, IL-12p70 according to the manufacturer’s instructions. Data was acquired on an LSRII and analyzed using FlowJo (TreeStar) and Prism. Briefly, mean fluorescence intensity for each cytokine was determined in FlowJo and experimental values were interpolated from the standard curve created using the BD CBA Mouse Inflammation Standards.

### Histology

Mice were inoculated intravaginally with 10^8^ Cc-Control, PBS or 20 μg LPS from *Salmonella enterica serotype Minnesota* as described above. Four hours later vaginal tissue was harvested and placed in buffered formalin. Fixed vaginal tissue was embedded in paraffin, cut into 7 μM slices, and mounted on slides. Tissue was deparafinized in xylene and re-hydrated in ethanol before staining with Harris hematoxylin and eosin Y solution. Images were acquired on EVOS cell imaging system (ThermoFisher).

### Caulobacter ELISA

Mice were inoculated biweekly intravaginally with 10^8^ *C*. *crescentus* Cc-Control for 2 months. Vaginal lavage fluid was collected and prepared as described above before inoculation and 67 days post-inoculation. Vaginal lavage fluid supernatant was diluted in blocking buffer containing 1% bovine serum albumin, 10% FBS and 0.05% Tween-20. An anti-RsaA antibody raised in rabbits (concentration: 14 mg/mL) was used in a limiting dilution to create a standard curve for quantification of antibody levels, with limit of detection of 14 pg/mL. Ninety-six (96) well Nunc plates were coated with 2 × 10^8^ Cc-Control diluted in carbonate buffer and incubated overnight at 4 °C. Plates were washed and blocked with 1% BSA. Diluted vaginal lavage fluid or anti-RsaA antibody was added to each well. After incubation at room temperature for 2 hours the plates were washed and 1:1000 anti-mouse IgG-HRP (Sigma) or 1:1000 anti-mouse IgM-HRP (Sigma) were added. A goat anti-rabbit HRP antibody (1:1000) was used for the standard curve. The plates were incubated at room temperature for 2 hours then 1:10 O-Phenylenediamine dihydrochloride (Sigma, 2 mg/mL) and 1:200 Urea hydrogen peroxide (Sigma, 40 mg/mL) were diluted in citrate phosphate buffer and added to each well. Twelve minutes later 25% H_2_SO_4_ was added and the OD_490_ was determined using a plate reader. The standard curve was used to interpolate the unknown values for antibody levels in vaginal lavage fluid.

### HSV-2 *in vivo* studies

Mice were given subcutaneous injection of 2 mg Medroxyprogesterone 17-acetate 4–5 days before infection with HSV-2. 10^8^ recombinant *C*. *crescentus* cells prepared as described above were mixed with 10^5^ pfu HSV-2 to a final volume of 20 μL and immediately inoculated intravaginally as described above. Vaginal lavage fluid was collected 24, 48 and 72 hours post-infection and prepared as described above. Mice were scored daily for disease progression using a 5 point scale: 1 - slight redness of external vagina; 2 - swelling and redness of external vagina; 3 - severe swelling and redness of both vagina and surrounding tissue and hair loss in genital area; 4 - genital ulcerations with severe redness, swelling and hair loss of genital and surrounding tissue; 5 - severe genital ulceration and hind limb paralysis. Mice were euthanized when they reached a score of 4.

### Plaque assays

Vaginal lavage fluid was serially diluted in MEM and a limiting dilution was prepared. Plaque assays were performed on confluent Vero cells as described above.

### Statistics

Statistical analysis was performed with Prism GraphPad software. As indicated, Student’s t test or ANOVA with correction for multiple comparisons were used as appropriate to evaluate the significance of differences between groups. Kaplan-Meier survival curves were compared by log rank tests. Unless otherwise indicated, presented statistics represent the comparison between HIV/HSV2 + Cc-Control and HIV/HSV2 + recombinant *C*. *crescentus*. A p value of <0.05 was considered significant.

### Data availability

Data is available from the corresponding author upon request.

## Electronic supplementary material

Supplementary Information
