# Supplementary material for: Generation of a Dual-Target, Safe, Inexpensive Microbicide that Protects Against HIV-1 and HSV-2 Disease
Source: Sci Rep. 2018 Feb 12;8:2786. doi: 10.1038/s41598-018-21134-1 (PMC5809452; doi:10.1038/s41598-018-21134-1)
Supplement: Supplementary file 1 — Supplementary Information [file 41598_2018_21134_MOESM1_ESM.pdf]

Generation of a Dual-Target, Safe, Inexpensive Microbicide that Protects Against HIV-1 and HSV-2 Disease

Christina Farr Zuend, John F. Nomellini, John Smit, and Marc S. Horwitz

Supplementary Information

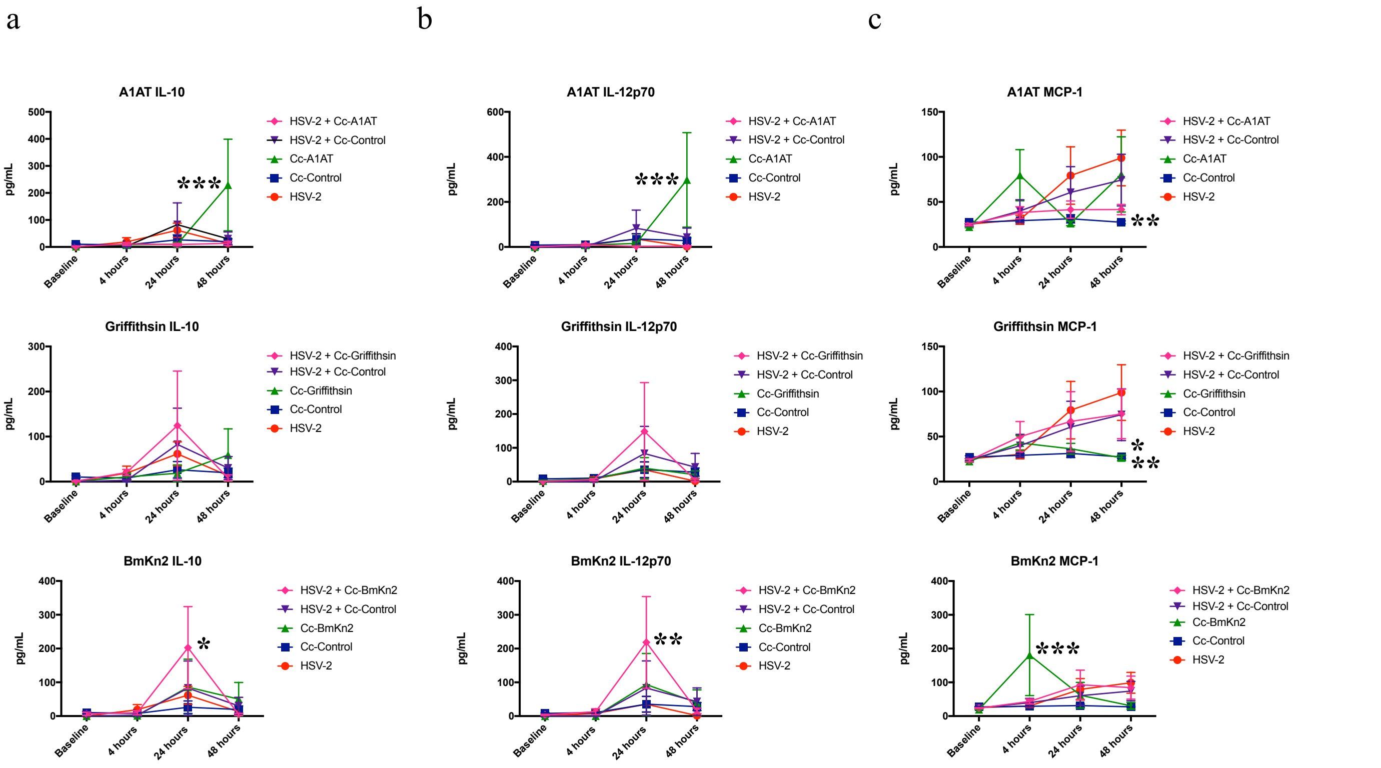

Supplementary Figure 1: Progesterone treated C57Bl/6 mice were infected intravaginally with 10^5 pfu HSV-2 strain 333 in the presence or absence of individual recombinant *C. crescentus* Cc-Control, Cc-BmKn2, Cc-Griffithsin or Cc-A1AT. Vaginal lavage fluid was collected prior to infection then 4, 24 and 48 hours post-infection and analyzed in a cytometric bead array for mouse inflammatory cytokines. **A)** IL-10 **B)** IL-12p70 **C)** MCP-1. N=4-11 mice per group. For each cytokine statistics were performed using two-way ANOVA with Bonferroni's correction for multiple comparisons. Presented statistics represent the comparison between HSV-2 and each experimental group. All other statistical comparisons were not significant. *p<0.05, ** p<0.01, ***p<0.001.
